# Whole-body CT characteristics of patients with severe fever with thrombocytopenia syndrome

**DOI:** 10.1007/s11604-026-02044-z

**Published:** 2026-07-01

**Authors:** Haruka Sato, Fumito Okada, Ryuichi Takenaka, Ryuzo Abe, Haruto Nishida, Yoshiki Asayama

**Affiliations:** 1 Department of Radiology, Faculty of Medicine, Oita University, 1-1 Idaigaoka, Hasama-machi, Yufu, Oita 879-5593 Japan; 2 Department of Radiology, Oita Prefectural Hospital, Oita, Japan; 3 Department of Emergency Medicine, Faculty of Medicine, Oita University, Yufu, Japan; 4 Department of Diagnostic Pathology, Faculty of Medicine, Oita University, Yufu, Japan

**Keywords:** Computed tomography, Severe fever with thrombocytopenia syndrome (SFTS), Tick-borne, Lymphadenopathy

## Abstract

**Purpose:**

To characterize the computed tomography (CT) features of severe fever with thrombocytopenia syndrome (SFTS) and to identify imaging features predictive of an adverse prognosis.

**Materials and methods:**

Thirty-one patients diagnosed with SFTS between July 2017 and August 2025 and who underwent whole-body CT (17 men, 14 women; mean age, 75.3 years; 24 survivors, 7 non-survivors) were retrospectively reviewed. CT findings of the chest, abdominal organs, and regional lymph nodes were assessed. Initial CT scans (n = 31) were obtained a mean of 3.8 days (range, 0–7) after symptom onset, and follow-up CT scans (n = 20) were performed in 17 patients a mean of 6.0 days (range 1–18) after the initial scan, giving a total of 51 examinations. Additionally, for three cases in which autopsy was performed, correlation analysis was performed between the CT findings and pathological findings.

**Results:**

On initial CT, the most frequent finding was regional lymphadenopathy (n = 30, 96.8%), most commonly involving inguinal (n = 17, 54.8%) and axillary nodes (n = 11, 35.5%). Perinodal fat stranding was observed in 29 patients (93.5%). The most common thoracic finding was ground-glass opacity (GGO) (n = 17, 54.8%), followed by bronchial wall thickening (n = 16, 51.6%) and interlobular septal thickening (n = 15, 48.4%). Among abdominal organs, splenomegaly was the most frequent finding (n = 25, 80.6%), followed by renal enlargement (n = 15, 48.4%). On follow-up CT, the most frequently observed changes compared with the initial CT were increased renal volume (n = 17, 85%) and increased hepatic volume (n = 14, 70%). GGO was significantly more frequent in non-survivors than in survivors.

**Conclusions:**

Characteristic CT findings of SFTS include regional lymphadenopathy with perinodal fat stranding, followed by splenomegaly and GGO. GGO may be an imaging finding predictive of an adverse prognosis.

## Introduction

Severe fever with thrombocytopenia syndrome (SFTS) is an emerging tick-borne infectious disease caused by severe fever with thrombocytopenia syndrome virus (SFTSV), a member of the genus *Bandavirus* in the family *Phenuiviridae*, which was first identified in China in 2011 [1]. Cases of SFTS were subsequently reported in Japan [2] and South Korea [3] in 2013. In recent years, SFTS cases have also been reported outside East Asia, indicating that the geographic distribution of this disease is expanding [4].

The disease is characterized by high fever, gastrointestinal symptoms, thrombocytopenia, leukopenia, and elevated liver enzyme levels. Neurological manifestations such as altered consciousness and seizures may develop during the clinical course, and severe cases can progress to disseminated intravascular coagulation (DIC) and multiple organ failure [3, 5]. SFTS exhibits high case-fatality rates in humans, despite supportive care (13–35%), and national surveillance data have shown that it is especially severe in people over 50 years of age, in which it has a higher fatality rate, posing a particular threat to Japan’s rapidly aging population [6, 7].

Although a few reports on the clinical and pathological features of SFTS have been published [1, 2, 8, 9], radiologic findings, particularly those on computed tomography (CT), have been less frequently reported. Although Ashizawa et al. described the chest CT findings of 22 patients with SFTS [10], to our knowledge, there have been no previous reports describing CT findings of the whole body. We therefore aimed to characterize the CT features of SFTS and to identify imaging features predictive of an adverse prognosis.

## Materials and methods

### Patients

Our institutional review board approved this study (3342) and provided a waiver for the requirement for informed patient consent because of the retrospective nature of the study.

We retrospectively identified 31 consecutive adult patients who were diagnosed with SFTS using an SFTSV polymerase chain reaction (PCR) test and who underwent whole-body CT (17 men, 14 women; age range, 65–89 years; mean age, 75.3 years; 24 survivors, 7 non-survivors) at our institution between July 2017 and August 2025 (Table 1). Patient characteristics were compared between the survivor and non-survivor groups.

**Table 1:** Patient characteristics and underlying conditions in the 31 patients

Characteristics	Total	Survivors	Non-survivors
	(n = 31)	(n = 24)	(n = 7)
*Age (years)*			
Range	65–89	65–89	69–79
Mean	75.3	75.6	74.3
M, F	17, 14	11, 13	6, 1
*Past history*			
Hypertension	11	9	2
Diabetes mellitus	10	5	5
Cardiac disease	7	6	1
Liver disease	2	1	1
Cancer	2	1	1
Hyperlipidemia	2	2	0
Collargen disease	2	1	1
Renal disease	1	0	1
Cerebral infarction	1	1	0
Interstitial pneumonia	1	1	0
Hyperuricemia	1	1	0
Pneumoconiosis	1	1	0
None	6	6	0

### CT examinations

All patients underwent thin-section CT using a multidetector CT system with 1-mm reconstruction, covering the region from the neck to the inguinal area, thereby including the cervical, axillary, and inguinal lymph node regions. CT examinations were performed with the patients in the supine position at full inspiration and were reconstructed using a high-spatial-frequency algorithm. Images were reconstructed using window settings that allowed viewing of the lung parenchyma (window level, − 600 HU; window width, 1500 HU) and the mediastinum (window level, 40 HU; window width, 400 HU). Abdominal images were reconstructed using a standard soft-tissue algorithm and were viewed using window settings appropriate for abdominal organs (window level, 40 HU; window width, 400 HU).

The initial CT examinations were performed 0–7 days (mean, 3.8 days) after the onset of symptoms. A follow-up CT examination was performed in 17 of the 31 patients (13 survivors and 4 non-survivors), at 1–12 days (mean, 5.1 days) after the initial CT in the 13 survivors, and at 2–3 days (mean, 2.5 days) after the initial CT in the non-survivors. A second follow-up CT was acquired in two of the survivors at 18 days after the initial CT, and in one of the non-survivors at 6 days after the initial CT. A total of 20 follow-up CT scans (15 in the 13 survivors and 5 in the 4 non-survivors) were acquired, all because of clinical deterioration. Intravenous contrast medium was administered in 11 of the 51 CT examinations.

In addition, postmortem CT (PMCT) was performed in four of the seven non-survivors, at 5–10 days (mean, 7.3 days) after the initial CT. All CT examinations performed (a total of 55 examinations) were included in the image analysis for this study.

### Imaging interpretation

Two radiologists (with 38 and 11 years of experience in CT image interpretation) who were unaware of the clinical outcomes interpreted the CT images independently, and conclusions were reached by consensus.

Lymphadenopathy was considered present when the short-axis diameter was > 10 mm or when there was clear asymmetry between both sides. The presence of perinodal fat stranding was also recorded. For the chest, each lobe was evaluated for the presence of ground-glass opacity (GGO), consolidation, interlobular septal thickening, bronchial wall thickening, nodules, and centrilobular nodules. Pleural effusion and pericardial effusion were also evaluated. For the abdomen, the presence of hepatomegaly, splenomegaly, pancreatic enlargement, and renal enlargement was evaluated. The volumes of the liver, spleen, pancreas, and kidneys were calculated using the SYNAPSE SAI Viewer (FUJIFILM Corporation, Japan). When automated measurements were deemed inaccurate based on visual assessment (e.g., mis-segmentation or inclusion of adjacent structures), manual corrections were performed. All corrected measurements underwent quality control, and the final values were determined by consensus between two investigators. For the liver and spleen, reference values were based on age-stratified mean volumes reported in healthy individuals by Hashimoto et al. and Harris et al., respectively [11, 12]. Pancreatic volume was assessed using sex-specific mean values derived from healthy adult populations as reported by Saisho et al. [13]. Renal volume was evaluated using mean values for adult men and women without renal parenchymal disease, as described by Cheong et al. [14]. Organomegaly was defined as a volume exceeding 1.2 times the corresponding mean reference value. These standard values are summarized in Table 2. In addition, gallbladder edema, perirenal edema, bowel wall edema, and subcutaneous edema were also evaluated. The frequencies of all CT findings throughout the body were compared between the survivor and non-survivor groups.

**Table 2:** Standard organ volume

	Mean ± SD (cm^3^)	Mean + 20%
*Liver (age, year)*		
< 30	1239.0 ± 221.9	1486.8
30–39	1205.2 ± 210.1	1446.2
40–49	1186.5 ± 225.4	1423.8
≧ 50	1110.8 ± 214.0	1333.0
*Spleen (age, year)*		
20–30	171.0 ± 57.8	205.2
31–40	132.4 ± 56.7	158.9
41–50	131.0 ± 83.0	157.2
51–60	119.3 ± 74.0	143.2
61–70	123.2 ± 43.4	147.8
71–85	89.2 ± 44.1	107.0
*Pancreas (adult)*		
Male	85.2 ± 26.9	102.2
Female	63.0 ± 20.5	75.6
*Kidney (adult)*		
Male	202 ± 36	242.4
Female	154 ± 33	184.8

SD, standard deviation

### Autopsy

Seven of the 31 patients died 5–14 days (mean, 9.9 days) after onset and 4–10 days (mean, 6.6 days) after the initial CT. Among them, autopsy was performed in three patients, 17, 100, and 137 h after the final CT. Each organ was examined histopathologically and the pathological findings were compared with the CT findings.

### Statistical analysis

Age, underlying diseases, and CT findings were compared between survivors and non-survivors. Age was analyzed using the Mann–Whitney U test, while underlying diseases and CT findings were analyzed using Fisher’s exact test due to the small sample size and expected cell counts of less than 5. All statistical tests were two-sided, and a *p*-value < 0.05 was considered statistically significant. Missing data were handled using complete-case analysis.

## Results

### Patients

The characteristics of all the patients are shown in Table 1. Hypertension was the most common underlying disease (11 patients, 35.5%), followed by diabetes mellitus (10 patients, 32.3%), cardiac disease (7 patients, 22.6%), liver disease (2 patients, 6.5%), and hyperlipidemia (2 patients, 6.5%). Six patients (19.4%) had no underlying disease. Diabetes mellitus was significantly more frequent in non-survivors (*p* = 0.04). No significant differences were observed between survivors (n = 24) and non-survivors (n = 7) with respect to age, sex, or other underlying diseases.

All non-survivors developed DIC and multiple organ failure. The immediate cause of death was respiratory failure in four patients, including acute respiratory distress syndrome (ARDS) in two and alveolar hemorrhage in two.

### Initial CT findings

**Fig. 1:**
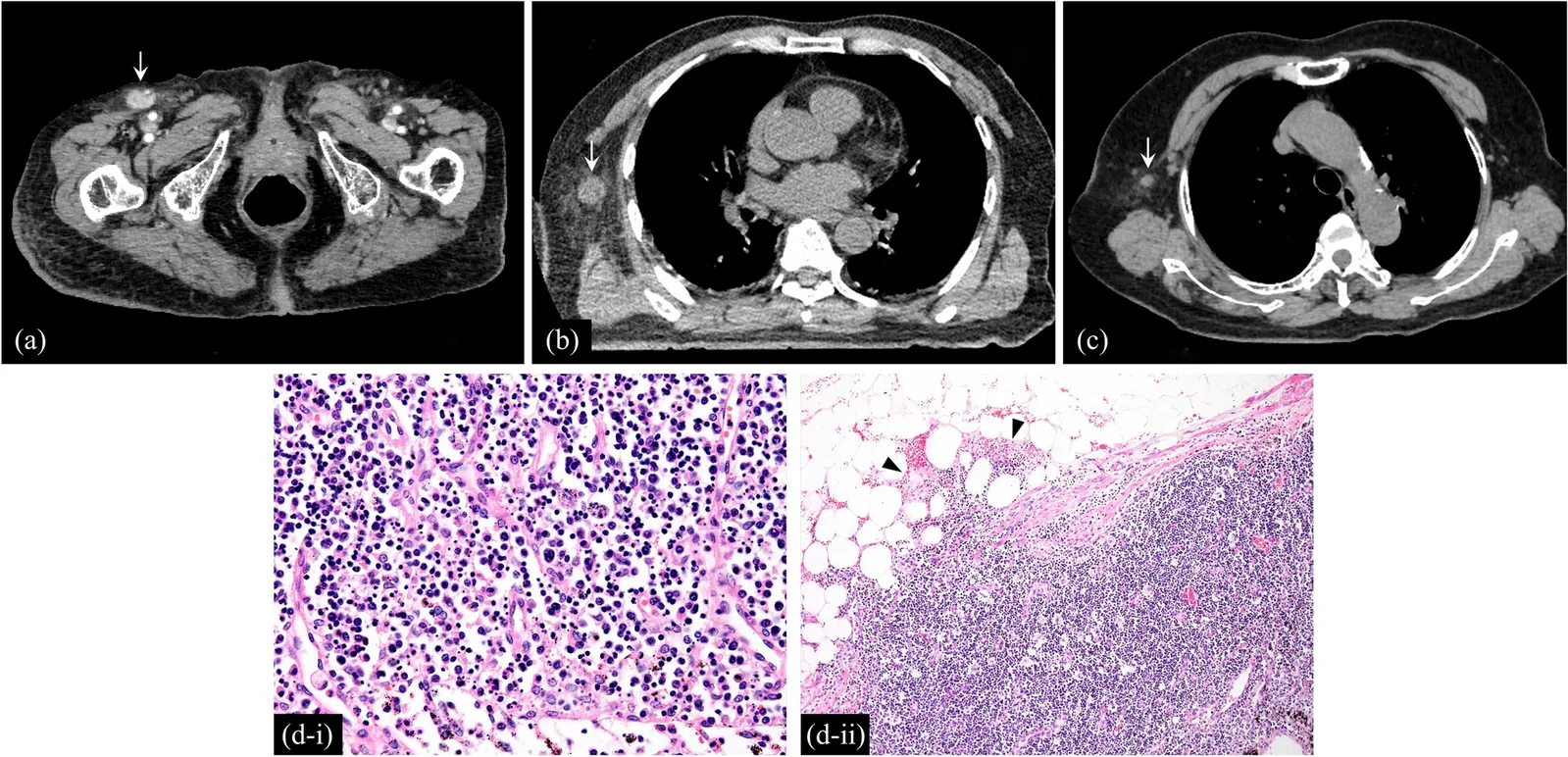
**a** Initial CT of SFTS in a 81-year-old female patient (survivor) 3 days after onset. Transverse enhanced CT image (1-mm thickness) at the level of the inguinal region shows a single enlarged right inguinal lymph node measuring 12 mm in short-axis diameter, accompanied with perinodal fat stranding (arrow). **b** Initial CT of SFTS in a 73-year-old male patient (non-survivor) 5 days after onset. Transverse unenhanced CT image (1-mm thickness) at the level of the right axilla shows a single enlarged right axillary lymph node measuring 13 mm in short-axis diameter, accompanied with perinodal fat stranding (arrow). **c** Initial CT of SFTS in a 72-year-old female patient (survivor) 4 days after onset. Transverse unenhanced CT image (1-mm thickness) at the level of the right axilla shows a small axillary lymph node measuring 6 mm in short-axis diameter, accompanied with perinodal fat stranding (arrow). **d** Histopathological findings of a lymph node (hematoxylin and eosin staining) in a 75-year-old male patient (non-survivor) 7 days after onset. (i) High-power photomicrograph (40 ×) shows lymphocytes being phagocytosed by histiocytes and undergoing apoptosis, with abundant nuclear debris. (ii) Low-power photomicrograph (10 ×) demonstrates inflammatory cells extending beyond the lymph node capsule, indicating extranodal inflammatory cell leakage (arrowheads)

CT findings of the 31 patients are summarized in Table 3. The most frequently observed abnormality on initial CT was lymphadenopathy, which was present in 30 patients (96.8%). The affected regions included the inguinal (17 patients, 54.8%), axillary (11 patients, 35.5%), and cervical (2 patients, 6.5%) regions. The size of the enlarged lymph nodes ranged from 7 to 19 mm in short-axis diameter (mean, 10.2 mm). All lymph nodes were predominantly round or oval in shape, and none demonstrated definite non-enhancing areas suggestive of internal necrosis. Perinodal fat stranding was observed in 29 of the 31 patients (93.5%) (Fig. 1a–c).

**Table 3:** CT Findings in the 31 patients

		Initial CT scan					Follow-up CT scan (n=20)							
							Second follow-up CT scan (n=17)				Third follow-up CT scan (n=3)			
		Total (n=31)	Survivors (n = 24)	Non-Survivors (n = 7)	*p* value		Total (n=17)	Survivors (n = 13)	Non-Survivors (n = 4)	*p* value	Total (n = 3)	Survivors (n = 2)	Non-Survivors (n = 1)	*p* value
Onset to initial CT (days)						Days from the initial CT								
	Mean (range)	3.8 (0–7)	4.0 (0–7)	3.3 (0.5)		Mean (range)		5.1 (1–12)	2.5 (2–3)			18 (18)	6 (6)	
Findings														
Chest CT findings														
GGO		17 (54.8)	10 (41.7)	7 (100)	0.0087		14 (82.4)	10 (76.9)	4 (100)	NS	2 (66.7)	1 (50)	1 (100)	NS
Consolidation		0 (0)	0 (0)	0 (0)	NA		3 (17.6)	2 (15.4)	1 (25)	NS	2 (66.7)	1 (50)	1 (100)	NS
Interlobular septal thickening		15 (48.4)	12 (50)	3 (42.9)	NS		11 (64.7)	8 (61.5)	3 (75)	NS	1 (33.3)	1 (50)	0 (0)	NS
Bronchial wall thickening		16 (51.6)	14 (58.3)	2 (28.6)	NS		11 (64.7)	9 (69.2)	2 (50)	NS	1 (33.3)	1 (50)	0 (0)	NS
Centrilobular nodules		0 (0)	0 (0)	0 (0)	NA		0 (0)	0 (0)	0 (0)	NA	0 (0)	0 (0)	0 (0)	NA
Nodule(s)		1 (3.2)	1 (4.2)	0 (0)	NS		0 (0)	0 (0)	0 (0)	NA	0 (0)	0 (0)	0 (0)	NA
No parenchymal abnormal findings		7 (22.6)	7 (29.2)	0 (0)	NS		3 (17.6)	3 (23.1)	0 (0)	NS	1 (33.3)	1 (50)	0 (0)	NS
Pleural effusion		10 (32.3)	9 (37.5)	1(14.3)	NS		12 (70.6)	8 (61.5)	4 (100)	NS	2 (66.7)	1 (50)	1 (100)	NS
Unilateral		3 (9.7)	3 (12.5)	0 (0)	NS		0 (0)	0 (0)	0 (0)	NA	0 (0)	0 (0)	0 (0)	NA
Bilateral		7 (22.6)	6 (25)	1 (14.3)	NS		12 (70.6)	8 (61.5)	4 (100)	NS	2 (66.7)	1 (50)	1 (100)	NS
Pericardial effusion		6 (19.4)	5 (20.8)	1 (14.3)	NS		8 (47.1)	5 (38.5)	3 (75)	NS	2 (66.7)	1 (50)	1 (100)	NS
Regional lymphadenopathy #		30 (96.8)	24 (100)	6 (85.7)	NS		16 (94.1)	13 (100)	3 (75)	NS	3 (100)	2 (100)	1 (100)	NA
Cervical		2 (6.5)	2 (8.3)	0 (0)	NS		0 (0)	0 (0)	0 (0)	NA	0 (0)	0 (0)	0 (0)	NA
Axillary		11 (35.5)	9 (37.5)	2 (28.6)	NS		4 (23.5)	4 (30.8)	0 (0)	NS	0 (0)	0 (0)	0 (0)	NA
Inguinal		17 (54.8)	13 (54.2)	4 (57.1)	NS		12 (70.6)	9 (69.2)	3 (75)	NS	3 (100)	2 (100)	1 (100)	NA
Perinodal fat stranding		29 (93.5)	24 (100)	5 (71.4)	0.045		15 (88.2)	12 (92.3)	3 (75)	NS	2 (66.7)	1 (50)	1 (100)	NS

Abdominal CT findings							Total	Survivors	Non-survivors	*p* value	Total	Survivors	Non-survivors	*p* value
Hepatomegaly		2 (6.5)	2 (8.3)	0 (0)	NS	Δ Liver Vol. *	13 (76.5)	9 (69.2)	4 (100)	NS	1 (33.3)	0 (0)	1 (100)	NS
Splenomegaly		25 (80.6)	19 (79.2)	6 (85.7)	NS	Δ Spleen Vol. *	10 (58.8)	7 (53.8)	3 (75)	NS	2 (66.7)	1 (50)	1 (100)	NS
Pancreatic enlargement		6 (19.4)	5 (20.8)	1 (14.3)	NS	Δ Pancreas Vol. *	11 (64.7)	8 (61.5)	3 (75)	NS	2 (66.7)	1 (50)	1 (100)	NS
Renal enlargement		15 (48.4)	14 (58.3)	2 (28.6)	NS	Δ Kidney Vol. *	15 88.2)	11 (84.6)	4 (100)	NS	2 (66.7)	1 (50)	1 (100)	NS
Gallbladder edema		2 (6.5)	2 (8.3)	0 (0)	NS		6 (35.3)	4 (30.8)	2 (50)	NS	2 (66.7)	1 (50)	1 (100)	NS
Perirenal edema		12 (38.7)	8 (33.3)	4 (57.1)	NS		11 (64.7)	7 (53.8)	4 (100)	NS	2 (66.7)	1 (50)	1 (100)	NS
Bowel wall edema		13 (41.9)	11 (45.8)	2 (28.6)	NS		8 (47.1)	4 (30.8)	4 (100)	0.029	1 (33.3)	0 (0)	1 (100)	NS
Subcutaneous edema		8 (25.8)	7 (29.2)	1 (14.3)	NS		10 (58.8)	8 (61.5)	2 (50)	NS	2 (66.7)	1 (50)	1 (100)	NS
Ascites		8 (25.8)	7 (29.2)	1 (14.3)	NS		9 (52.9)	7 (53.8)	2 (50)	NS	2 (66.7)	1 (50)	1 (100)	NS

^#^Lymphadenopathy was considered positive when the short-axis diameter was > 10 mm, or when there was clear asymmetry between both sides

*Δ Organ Vol. indicates an increase in organ volume compared with the initial CT scan

NA, not applicable (no variation between groups; all values were 0% or 100%); NS, not significant

**Fig. 2:**
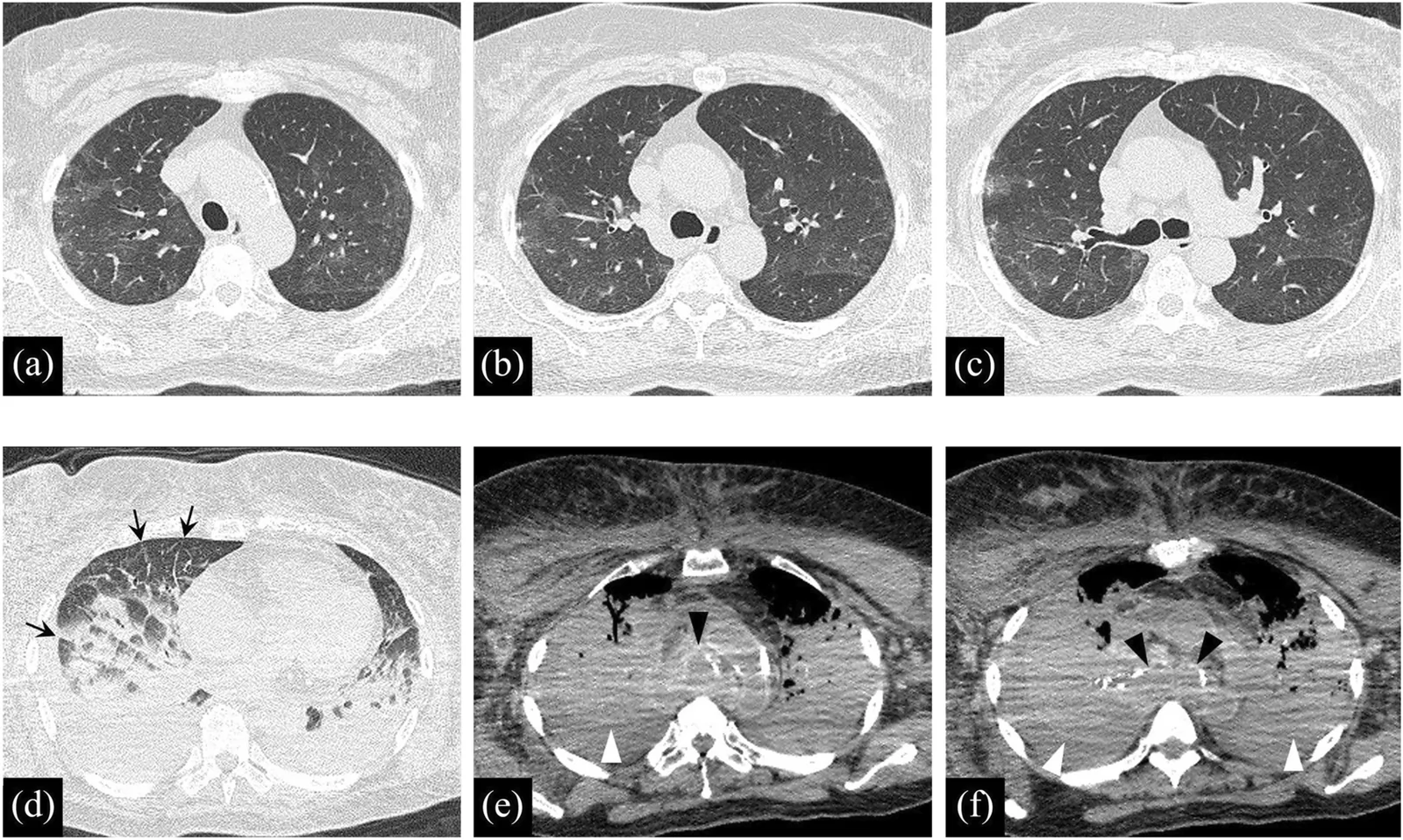
Initial CT (3 days after onset) and postmortem CT (PMCT) (10 days after onset) of SFTS in a 72-year-old female patient (non-survivor). **a**–**c** Initial CT: Transverse unenhanced CT images (1-mm thickness, lung-window setting) obtained **a** at the level of the aortic arch, **b** just below the aortic arch, and (c) at the level of the carina, show bilateral non-segmental ground-glass opacities (GGO) predominantly in the upper lobes. **d**–**f** PMCT: **d** Transverse lung-window CT image (1-mm thickness) at the level of the confluence of the right inferior pulmonary vein shows GGO and consolidation with interlobular septal thickening (arrows) in the right middle and lower lobes and left lower lobe. Transverse mediastinal-window CT images at the level of the trachea **e** and the carina **f** demonstrate high-attenuation material completely filling the trachea and both main bronchi (black arrowheads), consistent with blood clots. The lungs show diffuse increased attenuation suggesting massive pulmonary hemorrhage (white arrowheads), and bilateral pleural effusion is also present

The next most frequent abnormalities were pulmonary findings, which were seen in 24 of the 31 patients (77.4%). The most frequent pulmonary finding was GGO, which was present in 17 patients (54.8%) (Fig. 2a–c). This was followed by bronchial wall thickening in 16 patients (51.6%) and interlobular septal thickening in 15 patients (48.4%). Pleural effusion was observed in 10 patients (32.3%), with this being unilateral in three (9.7%) and bilateral in seven (22.6%). Pericardial effusion was seen in six patients (19.4%).

Regarding abdominal findings, splenomegaly was present in 25 patients (80.6%), renal enlargement in 15 patients (48.4%), bowel wall edema in 13 patients (41.9%), and perirenal edema in 12 patients (38.7%). Ascites was observed in eight patients (25.8%).

### Follow-up CT findings

**Fig. 3:**
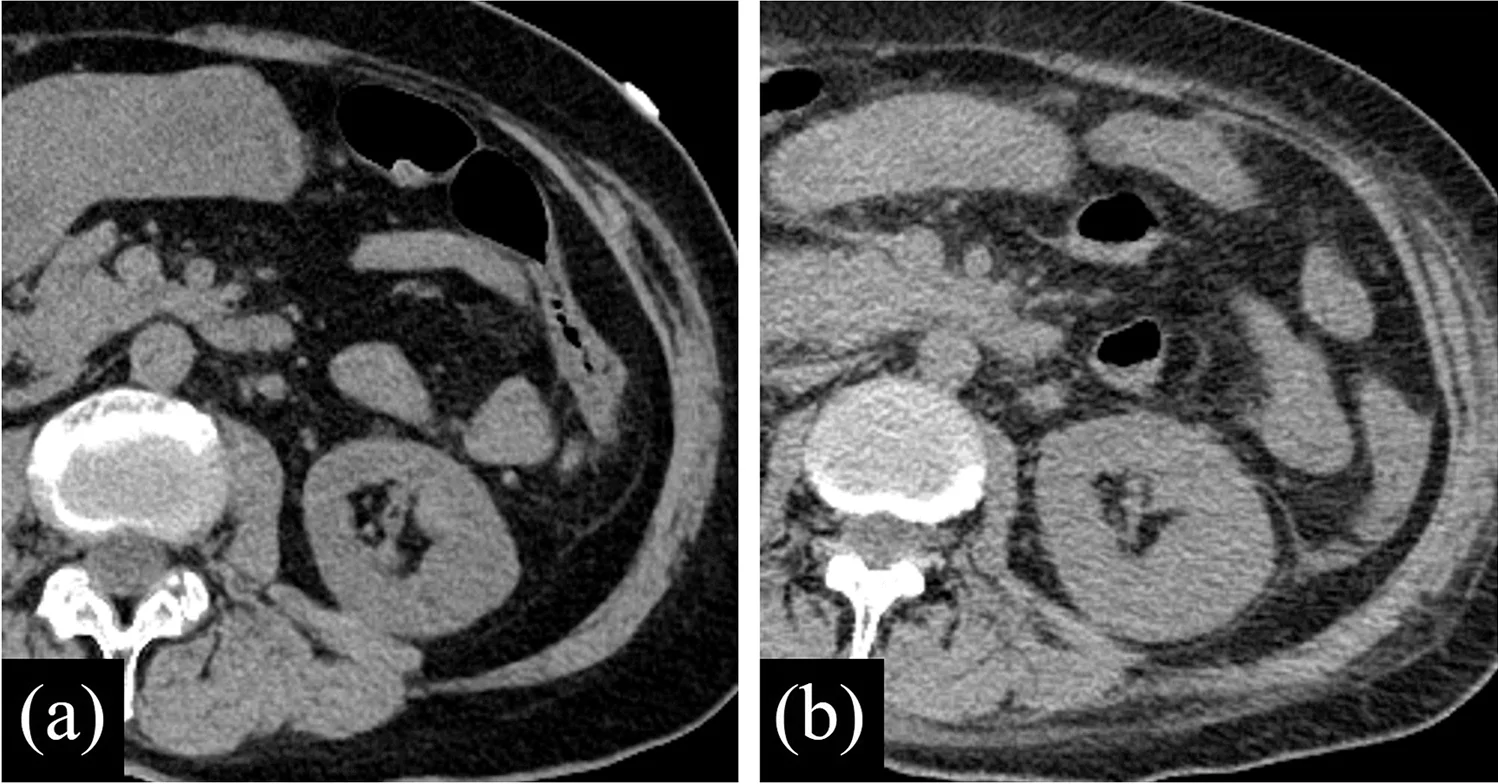
Serial abdominal CT of SFTS in a 68-year-old female patient (survivor) 6 and 24 days after onset. **a** Transverse unenhanced CT image at 6 days after onset shows no renal abnormalities. **b** Follow-up transverse unenhanced CT image obtained 24 days after onset demonstrates marked enlargement of both kidneys, with the total renal volume increasing from 316 to 508 mL

**Fig. 4:**
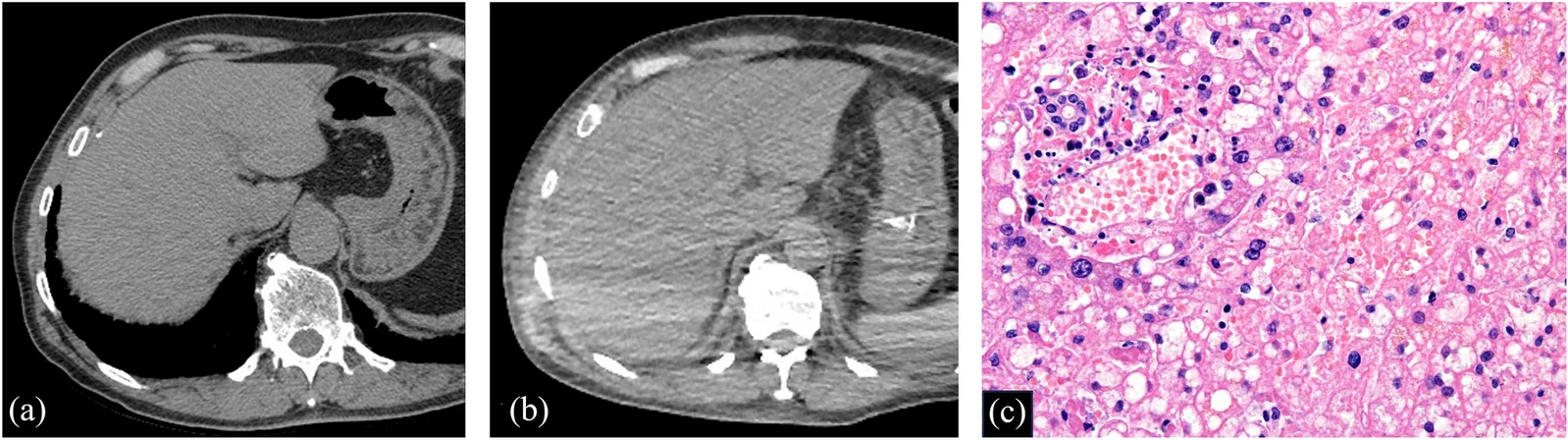
CT–pathologic correlation in a 79-year-old male patient (non-survivor). This patient is the same as that shown in Figs. 7 and 8, and died 12 days after onset. **a**, **b** Serial abdominal CT of SFTS obtained 5 and 11 days after onset. **a** Transverse unenhanced CT image obtained 5 days after onset shows no hepatic abnormalities. **b** Follow-up transverse unenhanced CT image obtained 11 days after onset demonstrates marked hepatomegaly, with the liver volume increasing from 768.9 mL to 1481.0 mL. **c** Histopathological findings of the liver (hematoxylin and eosin staining, × 40) obtained 12 days after onset: High-power photomicrograph shows hepatocellular vacuolar degeneration and ballooning change

**Fig. 5:**
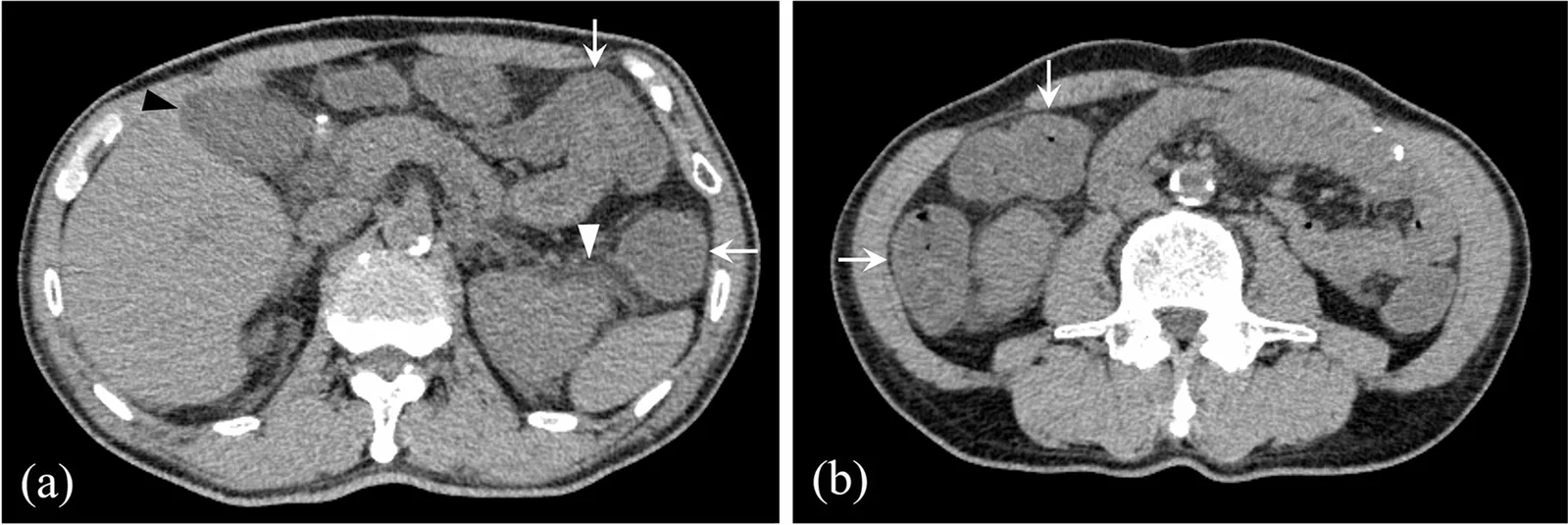
The second follow-up CT of SFTS in a 69-year-old male patient (non-survivor) 3 days after onset. **a** Transverse unenhanced abdominal CT image at the level of the origin of the superior mesenteric artery shows bowel wall edema (arrows) in the transverse and descending colon, as well as gallbladder edema (black arrowhead) and left perirenal edema (white arrowhead). **b** Transverse unenhanced CT image at the level of the lower pole of the right kidney demonstrates bowel wall edema involving the ascending and transverse colon (arrows)

On follow-up CT scans, the most frequently observed change compared with the initial CT was enlargement of abdominal organs. When the second CT (n = 17) scans were compared with the initial CTs, increased organ volume was observed in the kidneys on 15 examinations (88.2%) (Fig. 3), in the liver in 13 (76.5%) (Fig. 4a, b), in the pancreas in 11 (64.7%), and in the spleen in 10 (58.8%). Perirenal edema, bowel wall edema, gallbladder edema, and subcutaneous edema were observed in 64.7%, 47.1%, 35.3%, and 58.8% of examinations, respectively, with all of these being more frequent on the second scan than on the initial CT (Fig. 5). Ascites was present in nine examinations (52.9%).

Regarding lymphadenopathy, which was the most frequent finding on initial CT, no obvious changes in the number or size of enlarged lymph nodes were found on follow-up CT (n = 20) compared with the initial CT. Perinodal fat stranding also showed no clear changes on follow-up CT compared with the initial CT.

**Fig. 6:**
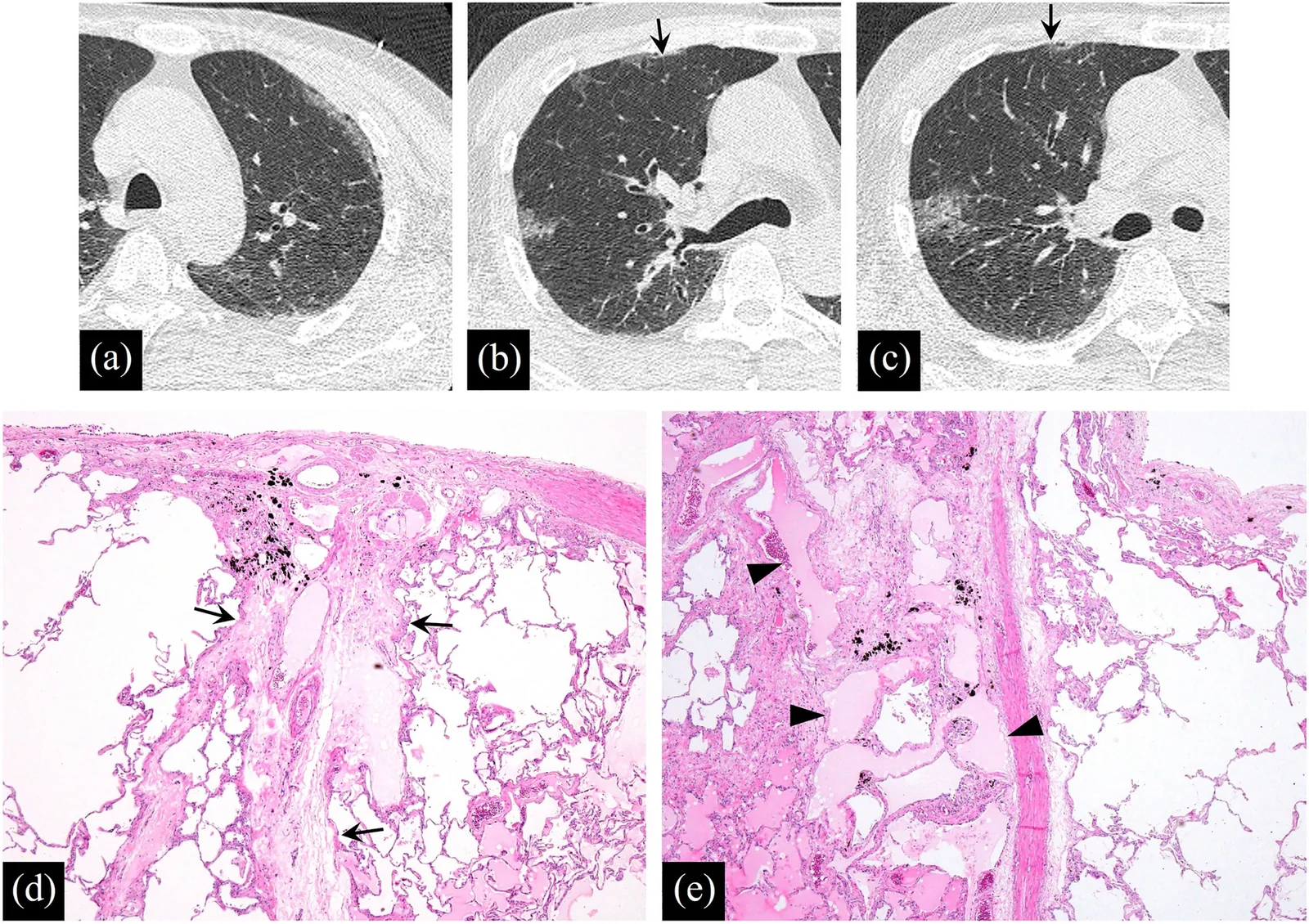
CT–pathologic correlation in a 70-year-old male patient (non-survivor). **a**–**c** Chest CT obtained 5 days after onset: Transverse unenhanced CT images (1-mm thickness, lung-window setting) obtained (a) at the level of the aortic arch show non-segmental ground-glass opacity (GGO) in the peripheral portion of the left S^3^. **b** At the level of the right main bronchus, CT demonstrates GGO with interlobular septal thickening (arrow) in the peripheral region of the right S^3^. **c** At the level below the carina, GGO and interlobular septal thickening (arrow) are observed in the right S^3^. **d**, **e** Histopathology (hematoxylin and eosin staining, 4 ×) obtained 11 days after onset: Low-power photomicrographs show interlobular septal thickening (arrows) and intra-alveolar proteinaceous exudates, consistent with non-cardiogenic pulmonary edema (arrowheads)

**Fig. 7:**
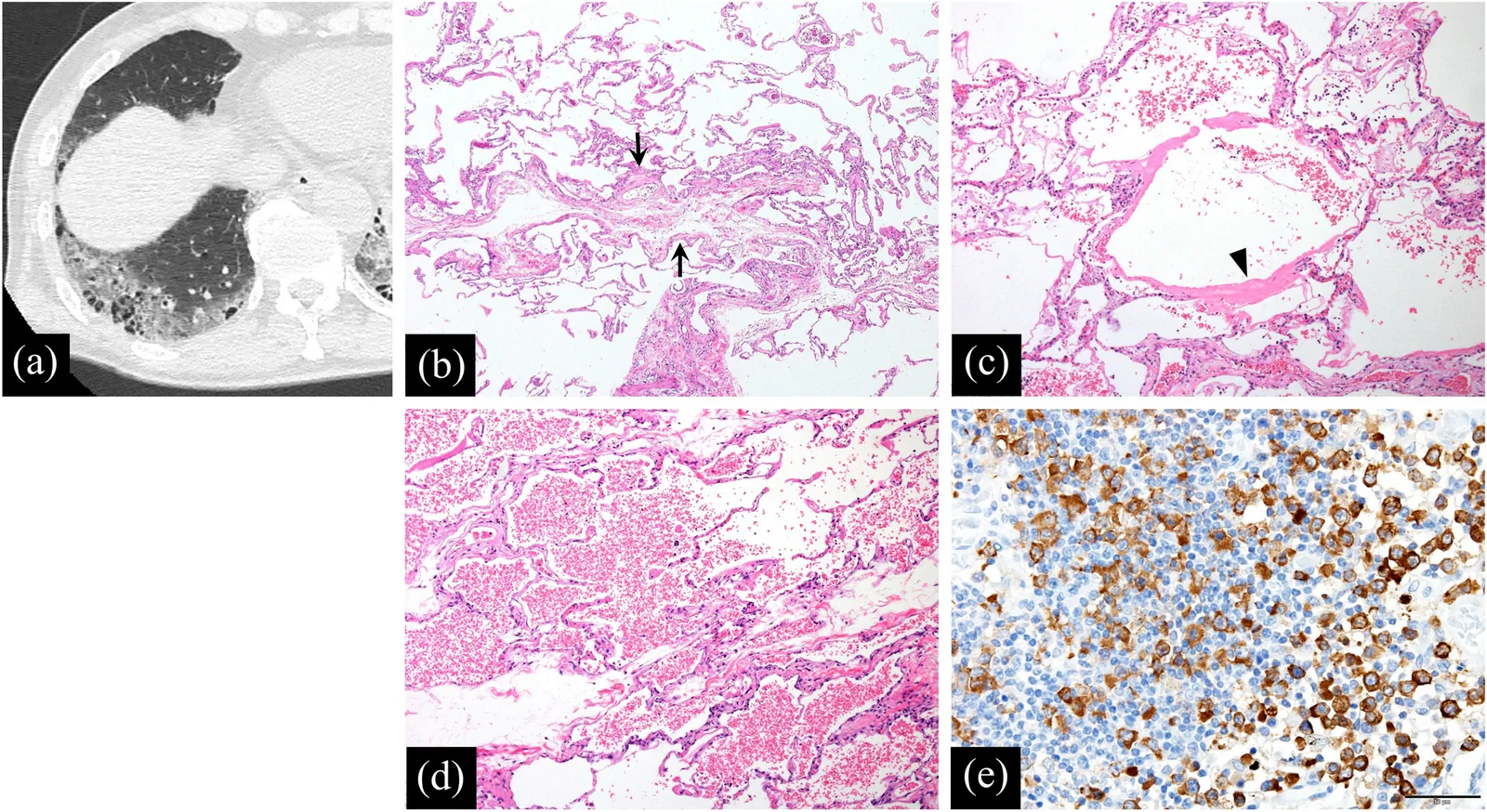
CT–pathologic correlation in the same 79-year-old male patient (non-survivor) shown in Figs. 4 and 8. **a** The second follow-up chest CT obtained 7 days after onset: Transverse unenhanced CT image (1-mm thickness, lung-window setting) shows non-segmental ground-glass opacity (GGO) in the peripheral region of the right lower lobe. **b**–**e** Histopathology obtained 12 days after onset: **b** Low-power photomicrograph (4 × , hematoxylin and eosin staining [H&G]) demonstrates thickening of the alveolar septa (arrows). **c** Intermediate-power photomicrograph (10 × , H&E) shows hyaline membrane formation (arrowhead), consistent with the exudative phase of diffuse alveolar damage (DAD). **d** Higher-power photomicrograph (20 × , H&E) reveals alveolar spaces filled with red blood cells, indicating alveolar hemorrhage. **e** High-power photomicrograph (40 × , immunohistochemistry using anti-SFTSV antibody) shows numerous SFTSV-positive infected cells

On follow-up CT, pulmonary parenchymal abnormalities were present in 16 of 20 examinations (80%). GGO was seen in all 16 of these, and bronchial wall thickening and interlobular septal thickening were each present in 12 examinations (60%), with these three findings all being found more frequently than on initial CT (Figs. 6a–c, 7a). Consolidation appeared in five examinations (25%) and pleural effusion in 14 examinations (70%), with the latter being bilateral in all cases. Pericardial effusion was observed in 10 examinations (50%). Both pleural and pericardial effusion were more frequent on follow-up CT than on the initial CT.

### PMCT findings

In all of the four PMCT examinations, the pulmonary findings included high-attenuation consolidation suggestive of hemorrhage, GGO, interlobular septal thickening, and bronchial wall thickening (Fig. 2d–f). High-attenuation fluid filling the trachea and bilateral main bronchi, presumed to represent hemorrhage, was also observed in all cases (Fig. 2e, f), as were bilateral pleural effusion and pericardial effusion.

In the abdomen, perirenal edema, bowel wall edema, and ascites were observed in all cases, and gallbladder edema was present in three patients (75%).

### Comparison of CT findings between survivors and non-survivors

On initial CT, GGO was observed in 10 of 24 survivors (41.7%) and in all seven non-survivors (100%), and was significantly more frequent in non-survivors (*p* = 0.0087). Perinodal fat stranding was also significantly more frequent in non-survivors (*p* = 0.045). There were no significant differences between the two groups in the frequencies or sizes of other pulmonary findings, abdominal organ abnormalities, or lymphadenopathy.

On follow-up CT, consolidation was observed in 3 of 15 examinations (20%) in survivors and in 2 of 5 examinations (40%) in non-survivors, and was more frequent in non-survivors, although the difference did not reach statistical significance. No other pulmonary findings differed significantly between the two groups. Among the abdominal organ findings on follow-up CT, bowel wall edema was significantly more frequent in non-survivors than in survivors (*p* = 0.029). No significant inter-group differences were observed in the other abdominal findings.

### Pathological findings

In the lymph nodes, lymphocytes phagocytosed by histiocytes were observed, and these lymphocytes had undergone apoptosis and necrosis. In addition, inflammatory cell infiltration extending beyond the lymph node capsule was observed, which was considered to reflect the perinodal fat stranding seen on CT (Fig. 1d).

In areas corresponding to GGO on CT, histopathology revealed non-cardiogenic pulmonary edema due to increased vascular permeability (Fig. 6d, e). Alveolar septal thickening and features of diffuse alveolar damage were also observed, along with alveolar hemorrhage (Fig. 7b–e).

**Fig. 8:**
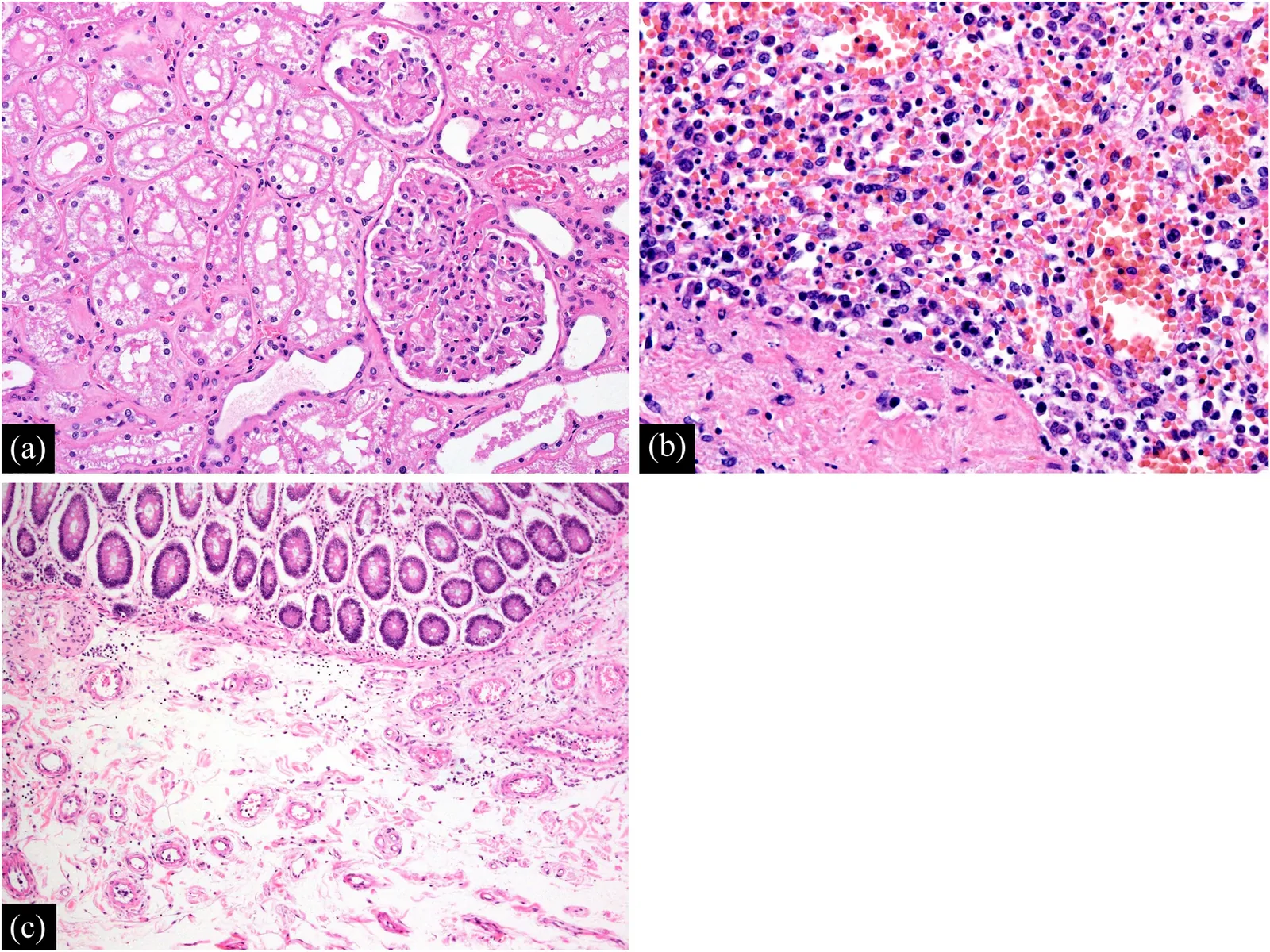
Histopathological findings of the kidney, spleen, and colon (hematoxylin and eosin staining, × 40) in the same 79-year-old male patient shown in Figs. 4 and 7. Histopathological specimens were obtained 12 days after onset, when the patient died. Corresponding CT images for **a**–**c** are not included in the manuscript. **a** Kidney: High-power photomicrograph demonstrates acute tubular injury characterized by epithelial simplification and cytoplasmic attenuation. **b** Spleen: High-power photomicrograph reveals lymphocytes being phagocytosed by histiocytes, with abundant apoptotic nuclear debris, consistent with hemophagocytosis. **c** Colon: Intermediate-power photomicrograph shows mucosal and submucosal edema within the bowel wall

In the liver, marked vacuolar degeneration and ballooning of hepatocytes were observed (Fig. 4c). Necrosis and hemorrhage were also present, together with widespread edema, with these being considered to be changes associated with DIC. In the kidneys, findings of acute tubular injury, congestion, and edema were observed (Fig. 8a). In the spleen, diffuse congestion was present, along with apoptotic lymphocytes, necrosis, and hemorrhage (Fig. 8b). In the pancreas and bowel, congestion and edema were observed, as in the other organs (Fig. 8c).

## Discussion

Following a tick bite, SFTS develops after an incubation period of 6–14 days, typically presenting with non-specific symptoms such as fever, fatigue, and diarrhea, with many patients initially presenting with fever of unknown origin. Only a minority of patients are aware of tick bites, and this proportion is lower than that reported for other rickettsial diseases such as scrub typhus or Japanese spotted fever [7]. Clinicians usually consider SFTS in the differential diagnosis on the basis of laboratory abnormalities such as leukopenia and thrombocytopenia. However, because characteristic symptoms are lacking and a clear history of tick bite is often absent, SFTS is often not suspected at an early stage.

SFTSV primarily infects immune cells such as monocytes and B cells. Previous reports have shown that the main target organs of SFTSV infection are the lymph nodes and spleen [9, 15, 16]. These secondary lymphoid organs are sites where mature lymphocytes recognize antigens and mount immune responses. SFTSV-infected cells have also been detected in many non-lymphoid organs, including the liver, intestine, lungs, and kidneys [15–17]. Infected cells release large amounts of cytokines (e.g., IL-6, TNF-α, IFN-γ), resulting in a “cytokine storm”. Excessive cytokines cause increased vascular permeability, leading to inflammatory cell infiltration into various organs and congestive or inflammatory enlargement of organs such as the liver and spleen [18].

In our study, the most frequent abnormal finding on initial CT was mildly-enlarged asymmetric regional lymph nodes accompanied by perinodal fat stranding. SFTSV is thought to spread from the tick bite site to a regional lymph node, where it triggers an immune response before disseminating systemically. If strong inflammatory reactions occur, one might expect more marked lymph node enlargement. However, in our study, although the involved lymph nodes were larger than those on the contralateral side, the mean short-axis diameter was only about 10.2 mm, indicating only mild enlargement. Previous reports on lymph node pathology in patients with SFTS have described necrotizing lymphadenitis as the main finding, with additional observations of hemophagocytosis and numerous apoptotic cells [15, 19, 20]. Our autopsy results showed similar histopathological features, including lymphocytes being phagocytosed by histiocytes and undergoing apoptosis. Thus, we suggest that although lymph nodes may initially enlarge because of inflammatory stimulation, concurrent phagocytosis, apoptosis, and necrosis of lymphocytes may prevent them from becoming markedly enlarged, resulting in only mildly-enlarged lymph nodes that “fail to become large”. Furthermore, histopathology revealed inflammatory cell infiltration extending beyond the lymph node capsule, suggesting “leakage” of inflammation into the surrounding tissue. This perifocal spread of inflammation likely corresponds to the perinodal fat stranding observed on CT. These features appear to represent imaging manifestations of a disease-specific pathophysiologic process in SFTS, which differs from that in other forms of infectious lymphadenopathy.

In patients with SFTS, the bite site is often not clearly recognized, and the eschar is small and difficult to detect on physical examination [7]. Therefore, identification of mildly-enlarged asymmetric lymph nodes with perinodal fat stranding on CT and reporting of them to clinicians may be extremely important for inferring the tick bite site and prompting early consideration of SFTS in patients presenting with fever of unknown origin.

To the best of our knowledge, the only published report comprehensively describing chest CT findings in patients with SFTS is that of Ashizawa et al. [10]. They reported chest CT findings in 22 patients with SFTS, with interstitial septal thickening and GGO the most common findings, being observed in 15 (68.1%) and 12 (54.5%) patients, respectively. Furthermore, they reported that GGO was associated with a poor prognosis. From the viewpoint of pathogenesis, they suggested that GGO in the lungs of patients with SFTS may be generated by lung edema [10] due to accelerated inflammation or disseminated SFTSV in the lungs. GGO, bronchial wall thickening, and interlobular septal thickening were also frequently observed in our study. On initial CT, GGO was present in 10 of 24 survivors (41.7%) but in all non-survivors, and was significantly more frequent in the non-survivor group (*p* = 0.0087), findings consistent with the previous report. On autopsy, GGO corresponded to non-cardiogenic pulmonary edema. In SFTS, a severe cytokine storm is induced, which is presumed to cause non-cardiogenic pulmonary edema through increased systemic vascular permeability [21]. Conversely, the presence of GGO on CT may indicate a severe cytokine storm and a marked host response. Therefore, the presence and extent of GGO may be useful indicators for assessing disease severity and predicting the prognosis in patients with SFTS. In addition, the alveolar septal thickening and diffuse alveolar damage observed in the autopsied lungs were consistent with previously reported pulmonary histopathology in SFTS, further supporting lung injury due to the cytokine storm [21].

To the best of our knowledge, there have been no previous reports describing abdominal CT findings in SFTS. In our study, initial CT revealed splenomegaly in 25 patients (80.6%), renal enlargement in 15 (48.4%), bowel wall edema in 13 (41.9%), and perirenal edema in 12 (38.7%). On follow-up CT, increased liver, spleen, pancreas, and kidney volumes were observed. In autopsied cases, these increases in organ volume were considered to be due to widespread edema resulting from increased vascular permeability associated with the cytokine storm, as well as inflammatory cell infiltration and necrosis. In the liver, hepatocellular vacuolar degeneration and ballooning also likely contributed to the increased volume. Hemorrhage was also present, and was considered to reflect changes associated with DIC. Diffuse congestion was observed in the spleen, with apoptotic lymphocytes, necrosis, and hemorrhage. The relatively mild increase in splenic volume compared with other abdominal organs may be attributable to lymphocyte apoptosis. On follow-up CT, bowel wall edema was more frequent in non-survivors than in survivors (*p* = 0.029). Histopathology demonstrated edema in the bowel wall, which was considered to reflect this CT finding.

SFTS is reported to cause coagulation abnormalities and DIC in severe cases, and DIC has been associated with a poor prognosis in several reports [3, 5]. Zhang et al. reported that DIC is a potential complication associated with high mortality in patients with SFTS, and emphasized the importance of early recognition and timely management of this serious complication. In a multi-center analysis including 34 institutions, Wang et al. reported that thrombocytopenia, coagulation abnormalities, liver dysfunction, and high viral load were associated with an increased risk of death, and that coagulation abnormalities suggestive of DIC were markers of severe disease. In our series of seven non-survivors, all patients developed DIC.

There are several limitations to our study. First, this is a retrospective study, and interpretation of CT images was performed by consensus. Second, the number of cases studied was small. Third, the dates of CT examinations and autopsies were not identical, with an interval of 4–10 days (mean, 6.6 days) between them. Fourth, correlation between CT and pathological findings could be performed in only three cases. However, all three autopsied cases demonstrated similar pathological findings. Fifth, the thin-section CT images were obtained using different protocols. Finally, the follow-up group included examinations performed at different time points and, in some cases, multiple scans from the same patient. Therefore, the analysis was conducted on a per-examination basis without accounting for within-patient correlation, which may have introduced dependency and temporal heterogeneity. In addition, the small number of third follow-up CT examinations may have limited the robustness of statistical comparisons.

In summary, in patients presenting with fever of unknown origin, even when a tick bite site cannot be identified, the presence of mildly-enlarged unilateral lymph nodes with perinodal fat stranding may serve as an important clue for early suspicion of SFTS. In addition, pulmonary abnormalities are frequently observable on chest CT, and GGO reflecting non-cardiogenic pulmonary edema may be an imaging finding predictive of a poor prognosis in patients with SFTS. However, given the small sample size, further validation in larger studies is required to confirm its prognostic value.

## References

[1] Yu X, Liang MF, Zhang SY, Liu Y, Li JD, Sun YL, et al. Fever with thrombocytopenia associated with a novel bunyavirus in China. N Engl J Med. 2011;364:1523–32. 10.1056/NEJMoa1010095 PMC3113718 21410387

[2] Saito T, Fukushima K, Umeki K, Nakajima K. Severe fever with thrombocytopenia syndrome in Japan and public health communication. Emerg Infect Dis. 2015;21:487–9. 10.3201/eid2103.140831 PMC4344266 25695132

[3] Zhang Z, Hu X, Du Q, Mo P, Chen X, Luo M, et al. Clinical characteristics and outcomes of disseminated intravascular coagulation in patients with severe fever with thrombocytopenia syndrome. BMC Infect Dis. 2025;25:508. 10.1186/s12879-025-10900-y PMC11992865 40217170

[4] Sansilapin C, Tangwangvivat R, Hoffmann C, Chaolek C, Lekcharoen P, Thippamom N, et al. Severe fever with thrombocytopenia syndrome (SFTS) in Thailand: using a one health approach to respond to novel zoonosis and its implications in clinical practice. One Health Outlook. 2024;6:18. 10.1186/s42522-024-00112-w PMC11443680 39350294

[5] Wang Y, Song Z, Wei X, Yuan H, Xu X, Liang H, et al. Clinical laboratory parameters and fatality of Severe fever with thrombocytopenia syndrome patients: a systematic review and meta-analysis. PLoS Negl Trop Dis. 2022;16:e0010489. 10.1371/journal.pntd.0010489 PMC9246219 35714138

[6] Kobayashi Y, Kato H, Yamagishi T, Shimada T, Matsui T, Yoshikawa T, et al. Severe fever with thrombocytopenia syndrome, Japan 2013–2017. Emerg Infect Dis. 2020;26:692–9. 10.3201/eid2604.191011 PMC7101122 32186502

[7] Kato H, Yamagishi T, Shimada T, Matsui T, Shimojima M, Saijo M, et al. Epidemiological and clinical features of severe fever with thrombocytopenia syndrome in Japan, 2013–2014. PLoS ONE. 2016;11:e0165207. 10.1371/journal.pone.0165207 PMC5077122 27776187

[8] Gai ZT, Zhang Y, Liang MF, Jin C, Zhang S, Zhu CB, et al. Clinical progress and risk factors for death in severe fever with thrombocytopenia syndrome patients. J Infect Dis. 2012;206:1095–102. 10.1093/infdis/jis472 22850122

[9] Takahashi T, Maeda K, Suzuki T, Ishido A, Shigeoka T, Tominaga T, et al. The first identification and retrospective study of severe fever with thrombocytopenia syndrome in Japan. J Infect Dis. 2014;209:816–27. 10.1093/infdis/jit603 PMC7107388 24231186

[10] Ashizawa H, Yamamoto K, Ashizawa N, Takeda K, Iwanaga N, Takazono T, et al. Associations between chest CT abnormalities and clinical features in patients with the severe fever with thrombocytopenia syndrome. Viruses. 2022;14:279. 10.3390/v14020279 PMC8877260 35215872

[11] Hashimoto T, Sugawara Y, Tamura S, Hasegawa K, Kishi Y, Kokudo N, et al. Estimation of standard live volume in Japanese living liver donors. J Gastroenterol Hepatol. 2006;21:1710–3. 10.1111/j.1440-1746.2006.04433.x 16984594

[12] Harris A, Kamishima T, Hao HT, Kato F, Omatsu T, Onodera Y, et al. Splenic volume measurements on computed tomography utilizing automatically contouring software and its relationship with age, gender, and anthropometric parameters. Eur J Radiol. 2010;75:e97–101. 10.1016/j.ejrad.2009.08.013 19775843

[13] Saisho Y, Butler AE, Meier JJ, Monchamp T, Allen-Auerbach M, Rizza RA, et al. Pancreas volumes in humans from birth to age one hundred taking into account sex, obesity, and presence of type-2 diabetes. Clin Anat. 2007;20:933–42. 10.1002/ca.20543 PMC2680737 17879305

[14] Cheong B, Muthupillai R, Rubin F, Flamm SCD. Normal values for renal length and volume as measured by magnetic resonance imaging. Clin J Am Soc Nephrol. 2007;2:38–45. 10.2215/CJN.00930306 17699385

[15] Hiraki T, Yoshimitsu M, Suzuki T, Goto Y, Higashi M, Yokoyama S, et al. Two autopsy cases of severe fever with thrombocytopenia syndrome (SFTS) in Japan: a pathognomonic histological feature and unique complication of SFTS. Pathol Int. 2014;64:569–75. 10.1111/pin.12207 PMC4282027 25329676

[16] Kaneko M, Shikata H, Matsukage S, Maruta M, Shinomiya H, Suzuki T, et al. A patient with severe fever with thrombocytopenia syndrome and hemophagocytic lymphohistiocytosis-associated involvement of the central nervous system. J Infect Chemother. 2018;24:292–7. 10.1016/j.jiac.2017.10.016 29138019

[17] Li S, Li Y, Wang Q, Yu X, Liu M, Xie H, et al. Multiple organ involvement in severe fever with thrombocytopenia syndrome: an immunohistochemical finding in a fatal case. Virol J. 2018;15:97. 10.1186/s12985-018-1006-7 PMC5977472 29848330

[18] Suzuki T, Sato Y, Sano K, Arashiro T, Katano H, Nakajima N, et al. Severe fever with thrombocytopenia syndrome virus targets B cells in lethal human infections. J Clin Invest. 2020;130:799–812. 10.1172/JCI129171 PMC6994144 31904586

[19] Tsuru M, Suzuki T, Murakami T, Matsui K, Maeda Y, Yoshikawa T, et al. Pathological characteristics of a patient with severe fever with thrombocytopenia syndrome (SFTS) infected with SFTS virus through a sick cat’s bite. Viruses. 2021;13:204. 10.3390/v13020204 PMC7912689 33572914

[20] Sakai L, Mura S, Kuwabara Y, Kagimoto S, Sakurai M, Morimoto M, et al. Lethal severe fever with thrombocytopenia syndrome virus infection causes systemic germinal centre failure and massive T cell apoptosis in cats. Front Microbiol. 2024;14:1333946. 10.3389/fmicb.2023.1333946 PMC10796997 38249467

[21] Sun Y, Jin C, Zhan F, Wang X, Liang M, Zhang Q, et al. Host cytokine storm is associated with disease severity of severe fever with thrombocytopenia syndrome. J Infect Dis. 2012;206:1085–94. 10.1093/infdis/jis452 22904342

